# Supplementary material for: An R-Package for the Deconvolution and Integration of 1D NMR Data: MetaboDecon1D
Source: Metabolites. 2021 Jul 13;11(7):452. doi: 10.3390/metabo11070452 (PMC8305572; doi:10.3390/metabo11070452)
Supplement: Supplementary file 1 [file metabolites-11-00452-s001.zip › metabolites-1285331-supplementary.pdf]

# Supplemenatal Material: An R-package for the Deconvolution and Integration of 1D NMR data: MetaboDecon1D

Martina Häckl[1], Philipp Tauber[2], Frank Schweda[2], Helena U. Zacharias[3,4], Michael Altenbuchinger[5], Peter J. Oefner[1] and Wolfram Gronwald[1*]

[1]Institute of Functional Genomics, University of Regensburg, 93053 Regensburg, Germany.
[2]Institute of Physiology, University of Regensburg, 93053 Regensburg, Germany.
[3]Department of Internal Medicine I, University Medical Center Schleswig-Holstein, Campus Kiel, 24105 Kiel, Germany.
[4]Institute of Clinical Molecular Biology, Kiel University and University Medical Center Schleswig-Holstein, Campus Kiel, 24105 Kiel, Germany.
[5]Institute of Medical Bioinformatics, University Medical Center Göttingen, 37077 Göttingen, Germany.
* Correspondence: wolfram.gronwald@klinik.uni-regensburg.de

*Availability of the package*
The R package MetaboDecon1D is freely available for download under https://www.uni-regensburg.de/medicine/functional-genomics/staff/prof-wolfram-gronwald/software/index.html.

*Installation procedure for Windows*
1) The *MetaboDecon1D_0.1.0.tar.gz* file for the MetaboDecon1D package should be saved localy in the *R* folder of the Documents folder. This could be the following path: "C:/Users/Username/Documents/R/win-library/3.6".
2) The package could be then installed in RStudio with:

*install.packages("C:/Users/Username/Documents/R/winlibrary/3.6/MetaboDecon1D_0.1.0.tar.gz", repos=NULL, type="source").*
3) In addition, the package employs the package "readJDX" (version 0.5.41) which needs to be installed by the user beforehand.
4) Afterwards the package could be loaded with the *library(MetaboDecon1D)* command.

*Usage of MetaboDecon1D*
MetaboDecon1D comes with an extensive help function including an example spectrum to demonstrate all of its functions.
The input arguments for *MetaboDecon1D()* are an NMR spectrum, which needs to be in **jcamp-dx** format, and its corresponding file path. Additional input arguments (*number_iterations*, *range_water_signal*, *signal_free_region*, *smoothing_param*, *delta*, *scale_factor*) have a default value and could be changed optionally. Further information regarding these parameters are supplied in the help function of the package, which is available with the *??MetaboDecon1D* command. *MetaboDecon1D* is then executed with *MetaboDecon1D(filepath, filename).*
The output of the *MetaboDecon1D()* function is a list containing values like the x-positions of all picked peak triplets, the generated Lorentz curves, the normed MSE value, the integrals of each Lorentz curves, etc. (further information is provided with the help function of the package). Each output value is accessible with the $ symbol.
The generated Lorentz curves are saved in a matrix in which each row represents the intensity values for one Lorentz curve.
The MSE values describe the deviation of the sum of the generated Lorentz curves and the original spectrum.
The integral values of the Lorentz curves, i.e. the area under each Lorentz curve, are saved in a vector where each entry represents the value for one Lorentz curve.

After the execution of the *MetaboDecon1D()* function there are three additional functions available to plot the results:

*plot_triplets(deconv_result)* to plot the picked peak triplets for the analyzed spectrum.

*plot_lorentz_curves_save_as_png(deconv_result)* which plots the original spectrum and all generated Lorentz curves and saves the result as *.png* file under the file path (current working directory).

*plot_spectrum_superposition_save_as_png(deconv_result)* which plots the original spectrum and the superposition of all generated Lorentz curves and save the result as *.png* file under the file path (current working directory).

*Example data set*
With the package there is a human urine 1D NMR spectrum as example data set available. The data set can be investigated with the MetaboDecon1D package with the following code:

```
# Load package
library(MetaboDecon1D)

# Execution of MetaboDecon1D
result <-MetaboDecon1D("load_example_path",
"example_human_urine_spectrum.dx")

# Get results
# Generated Lorentz curves
result$lorentz_curves
# MSE value
result$mse_normed
# All integral values of all Lorentz curves
result$integrals

# Plot picked peak tripelts, generated Lorentz curves and superposition
of them
plot_triplets(result)
plot_lorentz_curves_save_as_png(result)
plot_spectrum_superposition_save_as_png(result)
```

The generated *.png* files of the additional functions of the example data set are saved under the installation path of the package. For windows this could be the following path: “C:/Users/Username/Documents/R/win-library/3.6/MetaboDecon1D/extdata”.
The generated *.png* files of own NMR data are saved under the file path of the data (e.g. if the desktop is the location of the analyzed NMR files, then the generated *.png* files are also saved on the desktop).

*Computation time*
The computation time for one human urine 1D NMR spectrum amounts to about 3 min for the execution of the *MetaboDecon1D()* function for 10 iterations on a standard PC.
